# Recommendations for Addressing Suicidal Ideation Precipitated by Levetiracetam in the Treatment of Seizure Disorder: A Case Report and Review of the Literature

**DOI:** 10.7759/cureus.87919

**Published:** 2025-07-14

**Authors:** Ishrath Zamani, Misha Khalid Khan, Ann Manzardo, Naveen Khanzada

**Affiliations:** 1 Psychiatry, Deccan College of Medical Sciences, Hyderabad, IND; 2 Pediatrics and Child Health, Aga Khan University, Karachi, PAK; 3 Psychiatry, University of Kansas Medical Center, Overland Park, USA; 4 Child Psychiatry, University of Kansas Medical Center, Overland Park, USA

**Keywords:** anti epileptics, levetiracetam, psychiatric disorders, recommendation, self-harm, suicidal ideation

## Abstract

Suicidal ideation with levetiracetam is a relatively common occurrence in psychiatry and neurology practices. However, there are no established treatment guidelines, and more empirical evidence is needed to guide medical management in these cases. This report describes the case of a 17-year-old male patient with a history of poorly controlled epilepsy on oxcarbazepine who developed worsening anxiety after the initiation of levetiracetam and presented with suicidal ideation for the past three weeks; this was effectively managed with comprehensive care in our inpatient psychiatric center, resulting in the resolution of these thoughts. To support this case, we conducted a literature review using MEDLINE (Medical Literature Analysis and Retrieval System Online), PubMed, and Google Scholar to explore existing treatment protocols for patients experiencing suicidal ideation related to levetiracetam. This study proposes a treatment protocol, presented as a flowchart, that outlines various treatment modalities based on assessed risk factors in patients with suicidality associated with levetiracetam. We conclude that establishing a psychiatric treatment protocol will enable providers to effectively manage an amenable risk factor of suicidality. There is a need for further evidence-based research studies to strengthen our proposed treatment protocol.

## Introduction

Suicidal ideation (SI) is the second leading cause of death among teens and early adults globally [1]. It is a complex and multifactorial phenomenon related to a variety of factors such as personal experience, mental and physical health [2-5]. Identified risk factors for suicide include prior psychiatric history, traumatic brain injury, substance use disorder, and psychosocial stressors, as well as certain illnesses [4]. People with epilepsy have a five-fold greater suicide risk than people without epilepsy [5]. This relationship is independent of the influences of antiepileptic drugs (AED) or the presence of co-morbid psychiatric disorders [6]. The incidence of comorbid emotional disorders and risk of depression is higher in patients with epilepsy compared with the general population, which also increases the risk of suicide [3]. The risk of depression is more significant among patients with uncontrolled seizures than in those who are seizure-free [6]. Of added concern, the risk of suicide in epilepsy has been shown to double with the use of anti-epileptic medications [5]. In 2008, the U.S. Food and Drug Administration published a warning after a meta-analysis of data and reported that suicidality occurred in 4.3 per 1,000 patients treated with AEDs in the active arm compared to 2.2 per 1,000 patients in the comparison arm [6]. Multiple studies have shown a significant association between the anti-epilepsy medication, levetiracetam (LEV), and increased SI in epilepsy [3-5,7-10].

LEV is a second-generation, broad coverage, anti-seizure medication that works through modulation of synaptic vesicle glycoprotein 2A (SV2A) to inhibit presynaptic calcium channels (N-type), decreasing neurotransmitter release and thus acting as a neuromodulator. This hinders impulse conduction across synapses and facilitates gamma-aminobutyric acid (GABA)ergic inhibitory transmission [11]. LEV may also impact seizure risk through a modulation of inflammatory mediators involving interleukin-1 beta (IL-1 β. Research indicates that as the concentration of LEV increases, there is a corresponding decrease in IL-1β levels. Notably, both reactive astrocytes and microglia exhibit significant immunoreactivity for IL-1β and interleukin-1 receptor subtype 1 (IL-1R1). LEV appears to play a role in reducing reactive gliosis and diminishing the expression levels of the IL-1β system in areas such as the hippocampus and the piriform cortex. These findings suggest that LEV may provide an anti-inflammatory effect, particularly targeting the IL-1β system in neuroglial cells within the context of epileptic brains [2]. IL-1β has a role in the neuronal cell death after traumatic, ischemic, excitotoxic, and seizure-induced brain injury [12-14]. IL-1β inhibits astroglial glutamate reuptake by IL-1RI, leading to increased extracellular glutamate, contributing to seizure activity [15-17]. IL-1β also increases the N-methyl-d-aspartate (NMDA) receptor activity through IL-1RI-mediated activation, leading to increased intracellular Ca2+ and prolonged seizures [18,19]. 

LEV has rapid and complete absorption, high oral (~100%) bioavailability, minimal hepatic metabolism, and is primarily eliminated by the kidneys. The superiority of the efficacy, tolerability, and ease of dosing of LEV in epilepsy is well documented for mono-therapy treatment of partial-onset seizures with or without secondary generalization. It is also used as an adjunctive treatment of myoclonic seizures associated with juvenile myoclonic epilepsy and primary generalized tonic clonic (GTC) [20].

As stated, the utility of this effective treatment is limited by the associated behavioral adverse effect of suicidality, which often forces the withdrawal of the medication [21]. Dose-dependent increases in suicidality are typically seen within four weeks of LEV use [6,9,11,12,22]. Several studies showed LEV-associated psychiatric symptoms and other CNS side effects attributed to the underlying antiepileptic mechanism of action on GABA [3,22-24]. Molokwu et al. reported two cases of LEV dose-dependent rage, aggressive, and suicidal tendencies at a dose of 500 mg twice a day [11]. Kaufman et al. reported a case implicating higher dose LEV in the development of affective disorders with suicidal behaviors [9]. The exact underlying pathophysiological mechanism of suicidal behaviour/ideation is unknown. However, elevations of α 2A-adrenoceptors, 5-HT1A, 5-HT2A serotonin receptors, and μ-opioid receptors in the post-mortem brains of suicide victims have been found [10]. The mechanism for the relationship between SI and LEV is also not known but has been linked to modulatory activity on α-amino-3-hydroxy-5-methyl-4-isoxazolepropionic acid (AMPA) receptors. The drug, brivaracetam, has a similar mechanism of action and binding profile to LEV with fewer behavioural side effects [24-28]. Brivaracetam binds more selectively to SV2A than LEV and has no activity on the AMPA receptor. Behavioral adverse events (BAEs) that occurred during previous LEV treatment improved in 57.1% of cases after switching to brivaracetam [29].

Considering the high risk of SI within this patient population, there is a need for empirical evidence to guide medical management in these cases. This report describes a case of SI secondary to LEV treatment, reviews the existing literature, and provides a treatment protocol with a flowchart specifying treatment modalities in patients with suicidality associated with LEV. CARE reporting guidelines were used for case report [30].

## Case presentation

A 17-year-old patient, with a past history of cerebral palsy, seizure disorder, hydrocephalus, status post ventriculoperitoneal (S/P VP) shunt, and intrauterine stroke, was admitted to our inpatient psychiatric facility in February 2022, due to suicidal ideation for three weeks. Treatment with LEV was started about two months ago, on December 11, 2021. The patient described worsening anxiety over these two months, and for the last three weeks, he developed thoughts of wanting to hurt himself and had the intent to use a kitchen knife. The patient also wrote a suicidal note personalized to family members and very close friends.

Seizure and medicine history

The patient began having seizures at four years of age, in the context of febrile illness. Seizures manifested as focal with right face and eye twitching, followed by right-sided upper limb involvement, which may secondarily generalize. He also had an ongoing aura of a seizure with no outward movement noticed by the family. The patient was initially treated with oxcarbazepine, but he was transitioned to oxcarbazepine XR (extended release) 1800 mg nightly due to poor compliance. Before admission, his average seizure frequency was one seizure every four to six months, with the last unprovoked seizure in October 2021. LEV was added to this treatment protocol with the goal of making him seizure-free. The patient was initiated on 250 mg LEV twice daily for one week on December 11, 2021, then increased to 500 mg twice daily. This dose was maintained since December. He was also on oxcarbazepine XR 1800 mg at bedtime, with seizure frequency at his baseline.

Psychiatric history

The patient was regarded as a “quiet kid” in middle school who participated in normal social and school-related activities, i.e., bowling, school band, playing music, and writing. The patient began to experience symptoms of social anxiety, starting in his sophomore year of school, when he felt nervous and unable to talk in small gatherings, feeling shaky and isolated; however, he was never formally diagnosed with social anxiety. The patient had a panic attack at a small gathering with family members in his home the day before his admission. He described having symptoms of chest tightness, nervousness, and a racing heart. He reported worsening anxiety over the prior two months, experiencing worries about the judgment of other people, and the future. He reported low self-esteem, which did not significantly affect his life or school, with deliberate effort. At the time of admission, his social anxiety had escalated to the point of affecting his daily life activities.

The patient did not report a depressed mood or any suicidal ideation in the past, except in the last three weeks. He reported that for the last three weeks, he had been very depressed with a "snowball effect" and had suicidal attempts involving intent and a plan about two times, but he did not take any action except breaking a hanger to see if that would cut his neck. He endorsed low mood, suicidal ideation, and a plan to overdose and cut his neck. He also researched suicidal methods and narrowed down his research. He denied any sleep difficulties, appetite problems, guilt, or regrets. His Patient Health Questionnaire-2 (PHQ-2) score was 5 (normal: 0-2), and Patient Health Questionnaire-9 (PHQ-9) score was 15 (normal: 0-4).

He denied any obsessive-compulsive disorder or eating disorder symptoms. He denied psychosis or manic symptoms. The patient did not have psychiatric hospitalizations, had never seen psychiatrists, or had been on psychotropic medications. The patient did not report substance use in the past. He reported a family history of a maternal grandmother with bipolar disorder and a paternal grandmother who attempted suicide. There was no history of trauma. He lived with his mother, stepfather, and two younger brothers and regularly saw his biological dad, who lived overseas. He was currently in the 12th grade. The mother also corroborated the above history and reported the patient's mood changes of feeling anxious, withdrawn, with fewer emotions, and a depressed mood. The mother confirmed some anxiety in the past, but no depression.

Treatment protocol

The patient was admitted as an inpatient for psychiatric treatment. Prior to admission, the Neurology Department recommended tapering off the LEV over three days and starting on zonisamide 100 mg nightly while continuing on oxcarbazepine XR 1800 mg nightly. The patient received the diagnosis of major depressive disorder (MDD), first episode, and social anxiety disorder. Sertraline was considered for social anxiety and MDD, along with combined cognitive behaviour therapy (CBT) and supportive and family therapy. The patient’s mother expressed concerns about the initiation of selective serotonin reuptake inhibitor (SSRI) therapy due to a black box warning of increased suicidal ideation, but agreed to as-needed hydroxyzine for anxiety and panic attacks. The patient’s suicidal ideation improved during inpatient hospitalization under this protocol. The patient received intensive CBT, spiritual, expressive, music, recreational, and family therapy during his stay in the hospital. The patient showed improvement with CBT, where he discussed coping skills, meditations, and muscle relaxation skills, and also tapered off LEV in three days.

## Discussion

A review of the literature was carried out using MEDLINE (Medical Literature Analysis and Retrieval System Online), PubMed, and Google Scholar with no time limitation. The bibliographic sources were selected using the following keywords alone or in combination: “levetiracetam”, “suicidal ideation”, “suicidality”, “Anti epileptics”, “psychiatric disorders”, “treatment”, “recommendation”. The resulting search yielded 3600 articles, which were curated based upon the following criteria: publication in the English language, human studies on LEV-associated suicidal ideation/behaviors, and their treatment. Clinical studies or case reports for patients of all ages, treated for post-LEV psychiatric symptoms and SI, were included. Experimental studies, human or animal studies with psychiatric symptoms due to antiepileptic or epilepsy associated with behavioral and pharmacological treatment, but not specific to LEV treatment, were excluded. After applying the eligibility criteria, a total of 10 clinical studies were retained for review.  Our review was utilized to establish a psychiatric treatment protocol with a flow chart specifying different treatment modalities in patients with suicidality associated with LEV. 

Recent evidence-based clinical studies have shown multiple risk factors associated with suicidal behaviour among epileptic patients. Park et al.'s study showed increased SI in patients taking LEV with additional risk factors like previous psychiatric history, febrile convulsions, status epilepticus, intractable epilepsy, static encephalopathy, and temporal lobe epilepsy. It concluded that the use of LEV is associated with a higher probability of suicide (OR 7.6, 95%CI 1.1-53.7) [31]. Moreover, other clinical studies have also suggested a dose-response relationship between LEV and suicide-related behaviour [11,12]. Our case report supports the previously published studies showing a clear relationship between the use of LEV and the development of SI. 

The PHQ-9 is an effective screening tool for major depressive disorders [32]. Mini International neuropsychiatric Interview and SAD PERSONS (S: male Sex, A: Age younger than 19 or older than 45, D: Depression, P: Previous suicide attempt, E: Excess alcohol or substance use, R: Rational thinking loss, S: Separated or Single, O: Organized plan, N: No social support, and S: Sickness) scales are also used for screening for suicide [33-34]. A recent public health perspective on psychosis screening has stated that we currently do not have an approved psychosis screening tool at the patient level, particularly in the age range of peak symptom onset. However, when taking patient history, it is essential to document all psychotic symptoms like hallucinations, unusual thought content, disruptions of thought process, distress, and their associated impact on patients’ personal life [33,34].

In a case report by Almazan et al., their patient on LEV developed severe anxiety and depression with a GAD-7 score of 17 and a PHQ-9 score of 20 [35]. LEV was considered the cause of exacerbation. The patient’s LEV 500 mg twice-daily dose was substituted with carbamazepine 200 mg twice daily. Lamotrigine was discontinued, and all other psychiatric medications were kept the same. At discharge, her GAD-7 score was 6 and her PHQ-9 score was 8. Similarly, our patient also improved after LEV was tapered off. Multiple other studies also saw an improvement when they switched from LEV to other AEDs like valproic acid, brivaracetam, and lamotrigine [25,36,37]. 

Patients on LEV are followed up using different assessment scores, namely, the Strengths and Difficulties Questionnaire (SDQ), Scale for Suicidal Ideation (SSI), Children’s Depression Inventory (CDI), and Quality of Life in Epilepsy (QOLIE). Vitamin D deficiency is common in patients taking LEV and valproic acid. Durá-Travé et al. saw an increased risk of vitamin D deficiency in patients on valproic acid monotherapy (OR: 1.9, 95%CI: 1.1-3.8) and LEV monotherapy (OR: 3.3, 95%CI: 1.5-7.5) [28,38]. Therefore, we propose that routine clinical testing of vitamin D levels could be beneficial in patients on levetiracetam, with subsequent supplementation recommended in patients who are found to be deficient [39]. 

Patient treated with LEV can have behavioral adverse effects like depressive mood, psychosis, anxiety, suicidal thoughts, irritability, aggression, and tantrum [40]. The Columbia and Yale Anti-Epileptic Drug Database Project included 4085 epileptic patients. Their study concluded that levetiracetam had a 22.1% rate of psychiatric and behavioural side effects (PBSEs), which was greater compared to other AEDs (P< 0.001, OR = 6.87). Furthermore, LEV showed a 17.7% intolerability rate, a 9.4% dose decrease rate, and an 8.3% complete cessation rate when compared with the aggregate of the other AEDs [41]. Marino et al.'s study showed pyridoxine as an additional therapy for patients receiving LEV to prevent BAEs [42]. In their study, 92% of patients on LEV were started on pyridoxine therapy after a month without stopping the levetiracetam treatment; behavioral adverse effects improved after 9.06 ±3.05 days of pyridoxine supplementation. No toxicity was reported during the study. Hence, we recommend using vitamin B6 supplementation to prevent LEV-associated BAEs (Table 1).

**Table 1:** Characteristics and conclusions of reviewed studies LEV: levetiracetam; AEDs: antiepileptic drugs; SDQ: Strengths and Difficulties Questionnaire; CDI: Children’s Depression Inventory; BAE: behavioural adverse effect; NDDI-E: Neurological Depressive Disorders Inventory for Epilepsy; HADSD: Hospital Anxiety Depression Scale; CBT: cognitive behaviour therapy; PST: problem-solving therapy

Study	Type	Psychosocial behavioural assessment	Previous psychiatric history	Results	Treatment modality used to treat suicidality
Mula and Sander, 2007 [10]	Retrospective chart review	Suicidal ideation with LEV	Yes (mood disorder)	Of 517 patients, 0.7% developed suicidal ideation	Withdrawal improved symptoms in a week.
Lee et al., 2011 [25]	Prospective, open-label study	-	Yes	Three (4.2%; two suicidal ideation and one attempt) out of 71 patients	Switching to lamotrigine/AEDs improved symptoms.
Bektas et al., 2017 [36]	Prospective case-control study	SDQ and CDI	No	15-year-old girl (baseline CDI:18) suicidal ideation and depression	Switching LEV to Valproic Acid (mood-stabilizing AED) at 1-month follow-up resolved symptoms.
Yates et al., 2015 [37]	Prospective study	QOLIE score (Quality of Life in Epilepsy Inventory-Form 31)	-	27/29 showed meaningful improvement in BAE (Mean change QOLIE-30-P total score of 12.1), improved HRQoL (health-related quality of life)	Switching from levetiracetam to brivaracetam
Grudet et al., 2014 [39]	Cross-sectional study	DSM of mental disorder, 25(OH)D2, 25(OH)D3 and IL-6 levels	Suicide attempters (n=59), non-suicidal depressed (n=17), Healthy controls (n=14).	Suicide attempters had low vitamin D and high IL-6	Vitamin D supplementation reduced IL-6 and lowered suicidality
Marino et al., 2018 [42]	Randomized, case-control, prospective study	-	-	Pyridoxine is safe and effective in controlling LEV-induced BAEs in children (p<0.001)	Patients were subdivided into two groups: LEV only (group 1) or LEV with supplemental pyridoxine (group 2)
Gandy et al., 2014 [47]	Prospective Study	NDDI-E	Yes	Significant improvement in NDDI-E (33) score (p=0.045), HADSD (p=0.048). Significant decrease in depression (p=0.014) and suicidal ideation (p=0.005) (39)	CBT for nine weeks in patients with epilepsy
Fitzpatrick et al., 2005 [44]	Prospective study	Suicidal ideation	-	Significant decrease in suicidal ideation and depression in N=110 participants	Benefits of a brief prevention intervention (video) based on the PST model.
Filteau et al., 1993 [45]	Double-blinded clinical drug trials	Hamilton rating scale for depression	Yes	Significantly more rapid and effective improvement in depressed mood and the lessening of suicidal ideation in 459 participants.	Treatment with specific serotonin reuptake inhibitors

**Figure 1:**
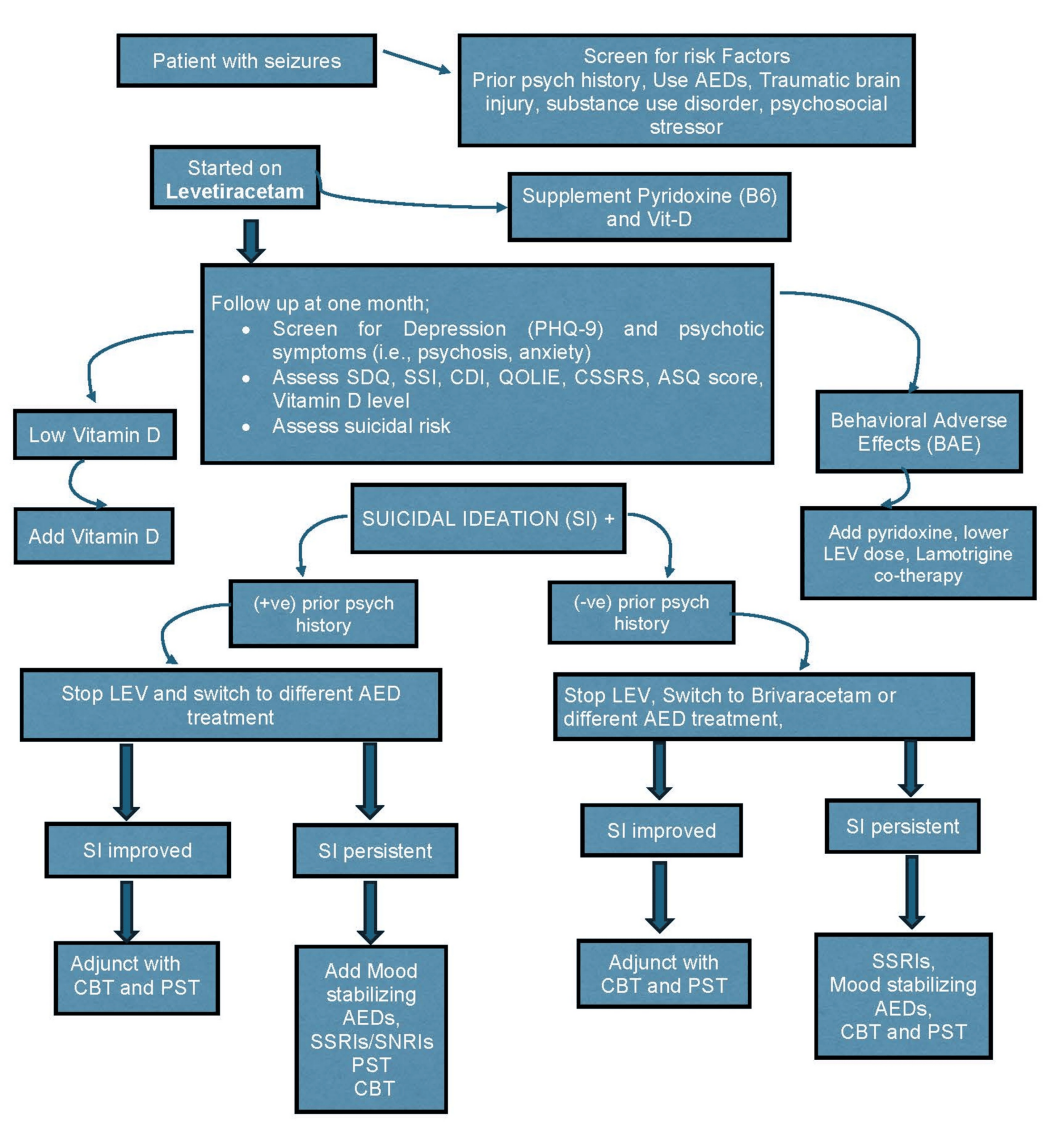
Treatment protocol in patients with suicidality associated with levetiracetam. SDQ: Strengths and Difficulties Questionnaire; SSI: Scale for Suicidal Ideation; CDI: Children’s Depression Inventory; QOLIE: Quality of Life in Epilepsy; CSSRS: Columbia Suicide Severity Rating Scale; ASQ: Ask Suicide-Screening Question; AED: antiepileptic drug; SSRI: selective serotonin reuptake inhibitors; CBT: cognitive behavioural therapy; PST: problem-solving therapy; SI: suicidal ideation; LEV: levetiracetam Mood stabilizing AEDs: valproate, lamotrigine, and oxcarbazepine

Our study aims to provide a treatment protocol given in the flowchart specifying treatment modalities in patients with suicidality associated with LEV. A patient with multiple risk factors for suicidality and taking LEV needs to be screened for depression using PHQ-9 and should be asked about their prior psychiatric history (Figure 1). It is recommended to stop LEV and use mood stabilizing AEDs (valproate, lamotrigine, and oxcarbazepine), CBT, and problem-solving therapy in patients with prior psychiatric history who agree to take AEDs. If SI persists, we can add an SSRI to the previous treatment regimen. On the other side, in patients with no prior psychiatric history who refuse to take AEDs, we recommend switching to brivaracetam, using CBT and PST. If SI persists, we can consider adding mood stabilizing AEDs or SSRIs to the previous treatment regimen. Our patient on LEV with SI was hospitalized, and LEV was tapered. Parents refused to give consent for SSRIs. The patient was effectively treated with as-needed hydroxyzine, intensive CBT, and spiritual, expressive, music, recreational, and family therapy during his stay in the hospital. Effective coping strategies were discussed. Our patient showed improvement and was later discharged with a follow-up plan. 

In the future, we would recommend conducting further clinical research studies on patients who developed SI while taking LEV. More extensive evidence-based clinical studies are needed to further strengthen our proposed treatment protocol [9,43-47]. In the meantime, physicians should effectively use different psychiatric assessment tools, check vitamin D levels, and follow up with patients in a timely manner to look for the development of any SI while taking LEV [29,38,39]. Any suicidal thoughts when taking LEV mandate immediate evaluation and triage to the appropriate level of care. Continual monitoring and use of evidence-based medical management for LEV-induced rage and suicidality by both caregivers and physicians can effectively address the potential morbidity and mortality.

## Conclusions

It is well known that suicide is a definite risk with epilepsy influenced by poor seizure control, concurrent depression, history of psychiatric illness, and affected independently by antiseizure medications, especially LEV. This warrants screening for depressive symptoms, inquiring about suicidal behaviors, evaluation by a psychiatrist for consideration of medication management for mood, anxiety, and BAEs, and psychotherapy tailored to the patient's needs, as well as counselling about potential side effects of increased suicidality, especially with antiseizure medications like LEV.
